# Ultrasound-guided interventions in elite soccer players

**DOI:** 10.1007/s00256-024-04801-5

**Published:** 2024-11-01

**Authors:** Gina M. Allen

**Affiliations:** 1 University of Oxford, Oxford, UK; 2 St Luke’s Radiology Oxford Ltd, Oxford, UK

**Keywords:** Soccer injuries, Ultrasound, Injections

## Abstract

**Abstract:**

In the world of elite soccer, or football as we call it in the UK, a player who cannot play for any length of time costs the team money and team performance. The time to return to play (RTP) is crucial in any player’s career, and the use of ultrasound-guided ultrasound injections has become important in the management of injury. In this article, I will explain the importance of good practice when performing these procedures and the use of steroids, sodium hyaluronic acid, platelet-rich plasma (PRP), and other therapies in achieving the goal of decreasing the time of RTP for the footballer.

**Key points:**

*•Injection therapies are routine practice in maintaining and treating soccer injuries.*

*•Injection therapies can be safely performed under ultrasound guidance.*

## Introduction

In today’s world, we can perform many injections to help the athlete continue their sport, whether in the short or long term. The implications of the soccer player not being able to play in a match at a premiership level are immense due to player salaries and team performance [1]. There is a discussion between the coach, physiotherapist, sports physician, and player. Not all injuries occur during competition, and training can cause injuries that need to be dealt with to get the player back to training.

The radiologist has become indispensable in providing a diagnostic service and guiding injections predominantly under ultrasound control.

Several new interventions are available, and the use of steroids is decreasing because of this. We should try and use evidence-based procedures but sometimes the literature is poor, as there are no good studies.

There are other imaging techniques used in the intervention, but it would be unusual to use these unless doing spine procedures, which is not covered in this article.

## Before starting the injection

Many of our medical indemnity organisations have exclusions regarding therapeutic injections in elite athletes. Usually, it is due to the referral pathway. The athlete should not be referred by the club coach, physiotherapist, or sports doctor, as these are not independent practitioners. Consider what sort of insurance you have in place. Some medical indemnity organisations will not indemnify you for treating elite athletes.

Discuss the pros and cons of the injection with the athlete. It is best to take and document that informed consent has been taken. This should include the following:

Verbal informed consent was obtained including the following warnings:

- Infection
- Allergy to the substance injected.
- Damage to vessels or nerves
- The injection may not relieve symptoms.
- Tendon rupture if near a tendon
- Potential side effects of steroids when used [2, 3]:
  o Headache
  o Facial flushing for approximately 48 h
  o Vivid dreams
  o Menstrual irregularity in women including intermenstrual bleeding and post-menopausal bleeding
  o Fat atrophy at the site of injection
  o Post injection flare
- Additional comment for injections in the region of the spine
  o Transient numbness and weakness in the limb due to local anaesthetic
  o Additional comments for soft tissue injections

A young person who plays sport and therefore is considered brave may be prone to fainting. This is a much more common occurrence in young males especially if they have tattoos. So lie the patient down if there are any concerns, and allow enough time for their recovery in your ultrasound room.

Consider the site of injection and the most appropriate injectate to use.

## Types of injection

### Steroid

Long-acting steroids are commonly used in the UK.

Triamcinolone and methylprednisolone are the most used in the musculoskeletal world. For more information, I would like to bring your attention to a good article by McMahon and colleagues [2].

Triamcinolone is a fat-soluble, fluorinated glucocorticoid, long-acting steroid that takes 48 h and is effective for 6 weeks. It is a derivative of hydrocortisone. It takes the forms of triamcinolone hexacetonide, triamcinolone acetonide, and triamcinolone diacetate, and acetonide is the most commonly used.

Other ingredients in the injectate are benzyl alcohol, polysorbate 80, carmellose sodium, and sodium chloride in water.

Five to 10 mg is needed for smaller joints and up to 40 mg for larger joints.

Methylprednisolone is a glucocorticoid derivative of hydrocortisone and prednisolone. It is water soluble. Other ingredients are sodium chloride, polyethylene glycol, myristyl-gamma-picolinium chloride, and water.

Four to 10 mg is needed for small joints, 10 to 40 mg for medium-sized joints, and 20 to 80 mg for large joints. The Achilles tendon does not have a tendon sheath, and injections of steroids in this region are regarded as hazardous.

Prior to administering a steroid, the patient’s drug history needs to be assessed as there are several interactions with other drugs.

Triamcinolone is the preferred drug in joints although has been linked with fat atrophy, and therefore, if a joint is close to the skin surface, most people would recommend methylprednisolone. I have however seen a case of fat atrophy after methylprednisolone, and therefore, this also can be a problem. The track of the injection should be broken up after removing the needle by agitating the soft tissue to help avoid this problem, but the patient should also consent, as it can leave a dent and white mark on the skin which can be permanent. It is much more common in patients with pigmented skin. Most patients however have some resolution by 18 months post procedure.

### Anaesthetic

Lidocaine is the most commonly used short-acting local anaesthetic. Its action occurs within 2 to 4 min of injection and provides local anaesthesia for up to 2 h post-injection. However, it has been linked to chondrotoxicity and should be avoided in joints if possible, especially in those with osteoarthritis [4].

Long-acting amide local anaesthetics include bupivacaine (Marcaine), ropivacaine (Naropin), and levobupivacaine, an enantiomer of bupivacaine (Chirocaine). The newer long-acting locals are found to be less chondrotoxic to joints. Still, the concentration is also a feature, so the lowest concentration possible should be used to decrease this problem [5, 6]. The newer agents are also less cardiotoxic. They can provide analgesia in as little as 20 min and last for approximately 12 h.

### Sodium Hyaluronic Acid

This is a naturally occurring glycosaminoglycan in synovial fluid and tendon sheaths. It is nature’s anti-inflammatory and lubricant, stimulating endogenous hyaluronic acid synthesis. It also provides a protective effect and decreases sensory nerve sensitivity. It was originally made from rooster combs (the highest occurrence in nature), but now, it is genetically engineered due to patients’ egg and chicken allergies.

It is formulated as a high (more than 500 to 2000 kDa), intermediate (800 to 1500 kDa), or low (less than 500 kDa) molecular weight substance. There are many products available. The rationale is that low molecular weight HA has little or no effect on the synthesis of endogenous hyaluronic acid, and a very high molecular weight hyaluronic acid suppresses the synthesis of endogenous hyaluronic acid; therefore, the intermediate-weight substances are the best according to this data [7].

Joint pseudo-sepsis occurred more commonly with high molecular weight formulations causing granulomatous synovitis in some studies [8].

Benzalkonium chloride skin preparations should not be used as this can cause precipitation of hyaluronic acid out of solution [9].

The size of the needle used to inject will depend on the MW of the product with high MW products needing an 18-G needle and intermediate MW products needing 21 G.

Often, there are other constituents including a phosphate-buffered sodium chloride and mannitol, which stabilises the chain of the hyaluronic acid and allows it to work for longer [10]. This allows a once-only injection [11]. If using an HA without mannitol, with a low molecular weight substance, up to 5 injections may be needed, and if using a high molecular weight substance, up to 3 injections may be needed.

This substance should be injected on its own without adding anaesthetic within the space of injection. It needs to be precisely injected as if it is not in a contained area, it will not benefit the tissue. The most successful areas of injection are therefore joints and tendon sheaths. It can be very painful if injected outside a contained area, especially if it is of a high molecular weight. Often, the patient feels pain at the site of injection for 48 h following it, due to a small amount of extravasation of the product.

It has a maximum effect for 8 weeks but a detectable effect for 24 weeks.

### Platelet-rich plasma (PRP) or autologous conditioned plasma (ACP)

This injection is obtained by taking 15 to 20 ml of venous blood and centrifuging it to extract the platelet-rich element without the red blood cells. The red blood cells make up approximately 45% which is forced to the bottom of the vial. White cells and platelets make up the middle layer called the buffy coat, which is approximately 1% of the blood volume, and the platelet-poor plasma (PPP) is the remaining top layer which is about 54% of the centrifuged blood [12].

Depending on the time and speed of rotation of centrifuging, a leucocyte-poor or leucocyte-rich platelet-rich plasma can be obtained. ACP is produced by a single centrifuge and uses all the buffy coat and PPP layer. PRP can be produced by a single or double spin method and extracts the buffy coat layer only. The use of white blood cells is unclear as these are suspected to inhibit healing, perhaps promoting scar tissue, inflammation, and damage to nearby tissues in vitro. The white blood cells may not aid healing but they are not thought to have any negative effect and may be beneficial in some other ways.

Approximately 5 ml of PRP will be obtained from 10 to 20 ml of venous blood. Some systems add an anticoagulant so the PRP can be injected after a longer time interval postproduction. These products need activation with additives such as thrombin, calcium chloride, or calcium gluconate which artificially activate the platelets and can stimulate clotting before use. PRP can also be stored, including freezing, and activated by additives when needed. The PRP is then injected in an area, after dry needling in most cases.

Anti-inflammatory medication should be avoided for the 1st 48 h after the injection so as not to inhibit the healing cascade.

### Prolotherapy

This is a procedure in which we use concentrated dextrose or sucrose to irritate tissues and cause cell proliferation. It was named after the shortening of “proliferation therapy”.

Ten percent of dextrose ruptures cell membranes osmotically, and phenol-glycerin-glucose causes cellular irritation and sodium morrhuate, which is an extract of cod liver oil, causes chemotactic attraction of cell mediators to initiate this reaction [13].

This treatment should be performed under local anaesthetic, and the patient warned that there may be increased pain around the area for up to 7 days following the procedure.

### Sclerosants

Polidocanol is used to sclerose vessels which are thought to be the reason for increased pain in tendinosis. The new blood vessels form when tendinosis occurs and bring pain fibres which cause an increase in pain [14]. This has been used extensively in Northern Europe where the experience exists in the Achilles and Patellar tendon [15, 16].

### Traumeel

It is said to be a multicomponent natural product containing 14 ingredients [17]. It is said to help healing by stimulating the release of anti-inflammatory cytokines and suppressing inflammatory mediators and is well tolerated. It is manufactured as a homoeopathy agent but contains some active ingredients. It does not have any side effects and no interactions with other medications. It is used much more commonly in Germany where it is manufactured, Italy, and Spain for muscle injuries and tendon and ligament problems.

## Procedures

### Dry needling

This is a procedure where the tissue is agitated using a cutting needle to make it bleed. Anecdotally, no more than 10 passes should be made. In the literature, it is also known as peppering, fenestration, and tenotomy. The patient should not be taking nonsteroidal anti-inflammatories either prior to or after the procedure, as this may negate the effects of the dry needling. This is a natural form of PRP, as blood products that clot at the site of the procedure are produced.

### Barbotage

This procedure has been performed in treating calcific tendinosis, especially in the shoulder. The use of warm saline to wash away acute calcification is performed by either using a 1-needle or 2-needle technique [18, 19]. It can be very effective in the acute phase of calcific tendinosis when the calcium is soft and liquid. In more established chronic tendinosis, the calcium is best broken up by a cutting needle as it cannot be washed away.

### Hydrodilatation

This procedure has gained favour in treating glenohumeral joint capsulitis or frozen shoulder. It involves introducing an amount of saline into the joint after the steroid injection. A total of 25 ml, including the other injectates, is enough to have a good response as was originally reported and is experienced in our practice [20]. Some people advocate a larger volume, but this will then rupture the capsule and may lead to compartment syndrome in the arm due to fluid extravasation down the arm.

### Saline stripping of tendons

**Fig. 1:**
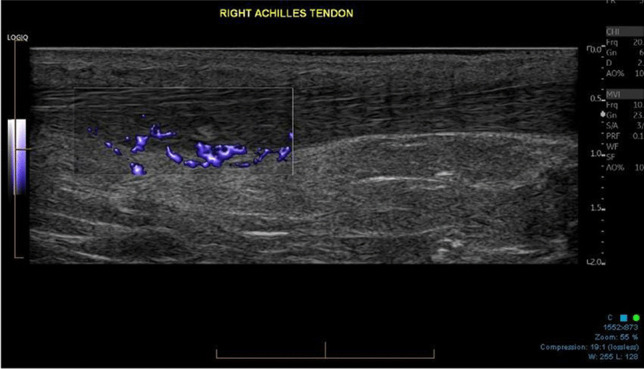
Ultrasound image of a midportion Achilles tendinosis with vascularity on MVI (microvascular imaging)

**Fig. 2:**
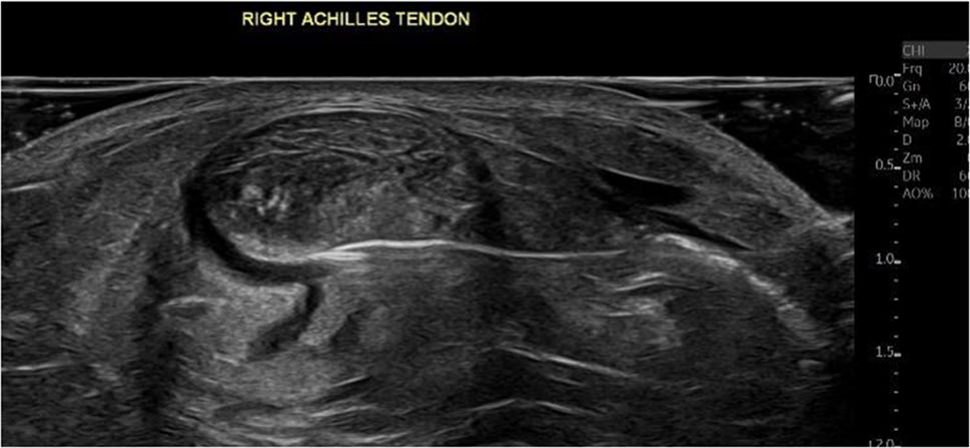
Ultrasound image of a needle inserted in front of the Achilles tendon to perform saline stripping of the tendon

**Fig. 3:**
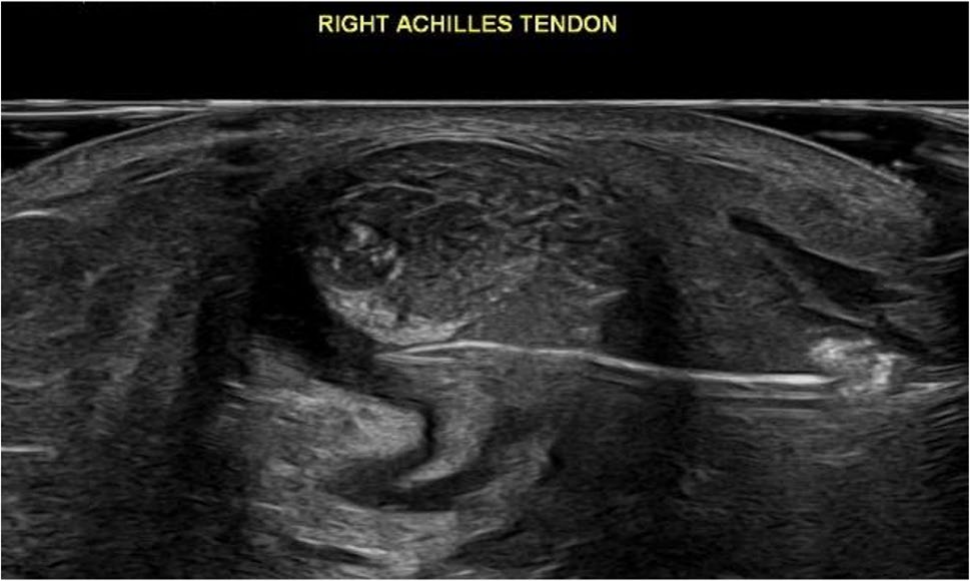
Ultrasound image of a needle inserted in front of the Achilles tendon to perform saline stripping of the tendon, now showing more fluid stripping the left edge of the paratenon

This is using saline to strip away the blood vessels from a tendon. Some authors advocate using a high volume with the addition of a steroid [21], but a good response can be obtained by using 25 ml, including local anaesthetic. It has been used extensively in the treatment of Achilles and patellar tendinosis when there is neovascularity Figs. 1, 2 and 3. Large volumes around the Achilles tendon can cause compartment syndrome.

## The procedure

The procedure itself should be performed in a clean environment and using an aseptic technique. If you need to know how to perform a procedure, I refer you to our book on ultrasound-guided MSK injections [22].

These injections should be done under ultrasound guidance watching the needle tip while performing the injection, to increase accuracy and decrease complications.

## Site of injection

### Muscle

The association football medical research programme audited injuries and found that the most common were due to muscle injury. If you included contusions, strains, and tears, then 44% of injuries were in muscle, and the majority were in the thigh [23]. This was also similar in the academy footballers (U9 to U21) with a 29% muscle injury rate, followed by ankle ligament injuries, with knee injuries peaking in the under 13 s, especially apophyseal injuries and hip and groin injuries in the under 15 s [24]. Most of these injuries occurred in training.

Involvement of the biceps femoris intramuscular tendon when the hamstrings are involved has a higher reinjury rate and much longer return to play than the other muscles [25]. Hamstring injury rates have also increased over the last 21 years in training and match play [26], and further research needs to be performed to explain why this has happened.

Standard treatment includes PRICE (protection, rest, ice, compression, and elevation) initially. The immediate treatment aims to reduce bleeding at the injury site, reduce pain, and minimise intramuscular oedema and scar tissue. A firm adhesive tape will help immobilise the area. Nonsteroidal anti-inflammatories should be avoided as they may decrease healing, but also, they will decrease the pain which may lead the player to use the limb more freely than is sensible. A programme of rehabilitation is important in early mobilisation, however [27]

## Intramuscular injections

### Aspiration of liquefying haematoma

It is proposed that muscles repair if the muscle fibres are close to one another with less blood components and cell debris to enhance the regeneration phase, so when you have a large haematoma, healing may be delayed. If you aspirate the liquefied blood, these fibres will unite more easily. The risk, however, is of introducing infection, so if performing this procedure, it needs to be done in a clean room with a sterile technique when the haematoma is truly liquid, so several days after injury. This treatment is performed widely in Europe [28], but I do not perform it in my practice.

#### PRP

Since our last discussion in our EJR article on injections in athletes [29], PRP continues to be used widely in muscle injuries.

In 2019, another paper showed that the use of PRP in grade 2 hamstring injuries impacted return to play. Those treated with PRP missed 22.4 days, 18.4 practices, and 1.7 games. Those who did not receive PRP missed 25.8 days, 22.2 practices, and 2.7 games [30]. Even though this means that the athletes returned one game sooner, this can significantly impact the team financially [30, 31].

### Traumeel

This compound is being used less frequently by most. There are no controlled trials to support its use in muscle, but some of the earlier articles compared its topical use with diclofenac gel in ankle sprains and found it was comparable in its pain relief [32].

## Tendon

### PRP

PRP appears to enhance healing by upregulating collagen type 1 gene expression and stimulating fibroblast and tenocyte proliferation, with the effect being greater in younger donors [33]. This should therefore have a positive effect on tendon and ligament injuries in a young athlete.

PRP and dry needling can improve function and pain in patellar tendinopathy [34]. Corticosteroid injections, high-volume injections, prolotherapy, sclerosing injections with polidocanol and HA are safe to treat patellar tendinopathy, but no studies are compare them, to say one is superior [34]. PRP is also used in the Achilles tendon [35] and lateral epicondylitis [36]. PRP can be used in the treatment of adductor-related pain, but there is conflicting evidence for its use [37].

### High-volume injections

The use of corticosteroid in addition to a high volume (HVI) stripping of a tendon is contentious, especially around the Achilles tendon when the increase of tendon rupture is seen due to the lack of tendon sheath and the major weight-bearing load taken by the tendon in normal activity. A double-blind study of 28 men treating midportion Achilles tendinosis compared using 50 ml of fluid with 10 ml 0.5% bupivacaine and normal saline with or without 20 mg of methylprednisolone under US guidance. All the subjects performed eccentric exercises after the injection. The VISA-A and VAS scores were better in the steroid group at 6 and 12 weeks post injection but not at 24 weeks, so there appears to be a better response in the steroid group in the short term [38]. The British National Formulary of Drugs and the National Institute of Clinical Excellence (NICE) advise that we should not use steroids next to the Achilles tendon. I have found that smaller injections of 25 ml without steroids can be very effective and do not run the risk of compartment syndrome.

### Prolotherapy

A meta-analysis of this treatment in chronic pain showed that prolotherapy is safe and can give greater pain relief when used with eccentric loading exercises for Achilles tendinosis than using this exercise alone. It has also been used in areas of mucoid degeneration in midportion Achilles tendinosis, directly targeting the affected area with dextrose, with a reduction of the size of these areas and reduced pain [39]. There are no controlled studies assessing its superiority to other treatments, however.

Dextrose prolotherapy has also been used with some success in chronic groin pain by injecting into the adductor tendon insertions [40].

Compared to steroids in lateral epicondylitis, prolotherapy was equal in effect at 3 months after injection [41].

Its use in rotator cuff tendinopathy showed no difference compared to steroid, PRP, or lidocaine, with an injection of each substance, randomly allocated, in 120 patients, giving a similar improvement in pain and function at 24 weeks in all groups [42].

### Steroid

It is not recommended for footballers, especially in weight-bearing tendons, unless the limb can be immobilised in a boot for 2 weeks following the injection.

### Dry needling

**Fig. 4:**
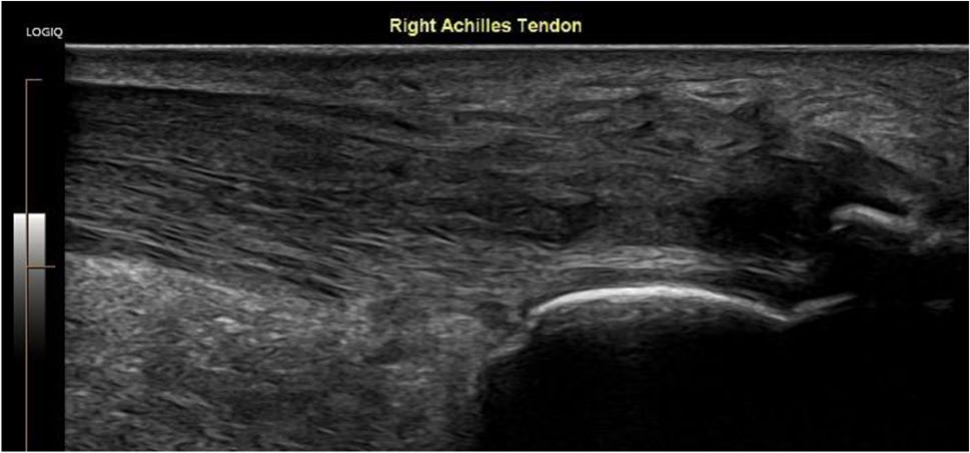
Ultrasound image showing an insertional Achilles tendinosis

**Fig. 5:**
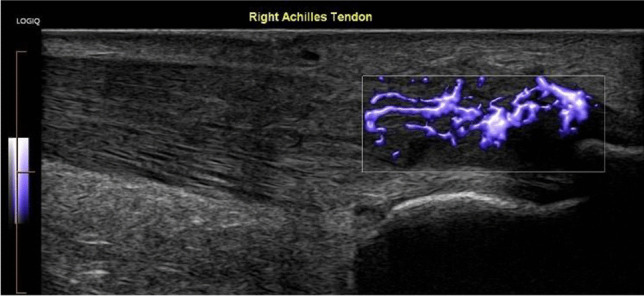
Ultrasound image showing an insertional Achilles tendinosis with vascularity on MVI (microvascular imaging)

**Fig. 6:**
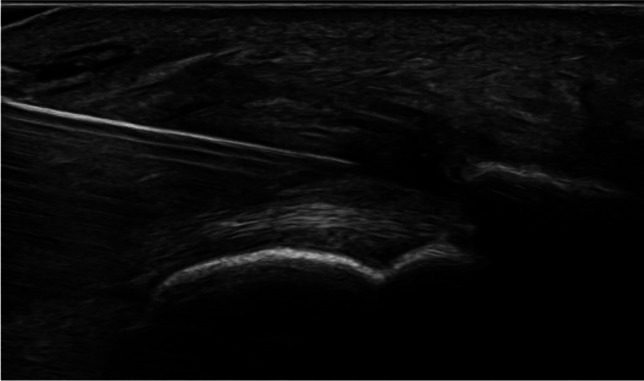
Ultrasound image showing dry needling of an insertional Achilles tendinosis

There is still no good research on dry needling alone although it is used extensively in clinical practice. A recent review still states that there appears to be positive outcomes from using this technique, but there is poor quality evidence [43]. In the literature, it is combined with PRP treatment. In clinical practice, I find it useful in insertional Achilles tendinosis, lateral epicondylitis, and insertional patellar tendinosis Figs. 4, 5 and 6.

The risks of tendon rupture are very low in these areas. Caution should be taken if using it in midsubstance Achilles tendinosis, and a longitudinal approach to the tendon should be adopted [44].

## Joint

### Steroid

A recent study from Nottingham looked at the likelihood of knee pain and total knee replacement after a career in Football concerning the use of injections (steroid, local anaesthetic, and others) versus knee injury. They found that 45% had an injection during their career, that approximately 70% had had steroid injections in their careers, and that some had had many injections (mean 7.5). Although most of these injections were after injury, 18% were not. They concluded that the more injections a footballer had received, the more likely they were to have continuing knee pain and total knee replacement [45].

**Fig. 7:**
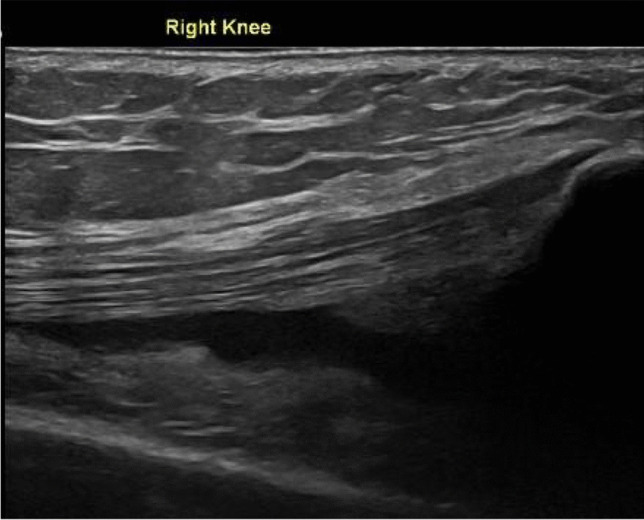
Longitudinal ultrasound image showing an effusion in a knee joint

**Fig. 8:**
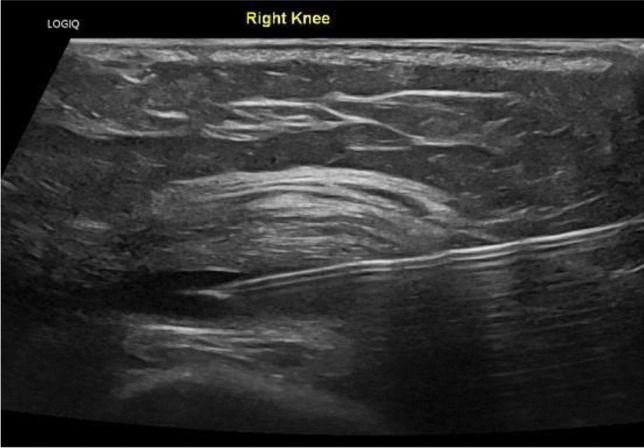
Ultrasound image showing a needle in the transverse position, under the quadriceps tendon, in an effusion in the suprapatellar pouch of the knee joint

Another paper showed some concern regarding cartilage attrition on MRI over a 2-year period in a group receiving steroid injections versus saline in the knee [46] Figs. 7 and 8. The above papers and steroids known chondrotoxicity should decrease their use in athletes [47].

### PRP

A meta-analysis by Chen et al. looked at 14 RCTs comparing PRP and HA and came to the conclusion that PRP was superior in pain relief and knee joint function but also admitted that there were different protocols used for the studies with different preparations of HA and PRP [48, 49]. A further study from the USA looked at 18 level 1 studies and found that PRP was superior in outcomes to HA and also noted that leucocyte-poor PRP may be superior to leucocyte-rich PRP [50].

PRP and prolotherapy are safe methods to treat osteochondral lesions of the talus, but the evidence is poor regarding efficacy [51].

### Sodium hyaluronic acid

A RC study from Italy compared high molecular weight HA and leucocyte-rich PRP injected for knee osteoarthritis (Kellgren-Lawrence grades 0 to 3) in 192 at weekly intervals for 3 weeks. Both treatments were just as effective but the need for further injections was reduced at 2 years in the PRP group [52].

## Ligament

### Steroid

Steroid is not routinely used in ligament injuries.

It can be used to relieve pain in lateral ankle ligament injuries when synovitis is present in the anterior lateral gutter.

It has also been used to good effect in athletes with persistent pain following a low-grade medial collateral ligament knee injury and deep MCL fibre scar tissue using triamcinolone injection and dry needling with immediate and sustained pain relief [53].

### PRP

Injection of PRP into a sprained anterior inferior tibiofibular ligament with a standardised rehabilitation protocol showed a decreased time to return to play and increased agility compared to a retrospective control group [54]. In an emergency department study, however, PRP plus lidocaine injected at the site of ankle injury compared to a control of saline injection, in a general population, showed no difference in pain relief or function at 30 days post injury [55].

Dry needling is often used as an adjunct to other treatments, such as the ones outlined above (PRP and steroid use).

### Prolotherapy

Prolotherapy is thought to stimulate healing and promote such in poorly vascularised tissues such as ligaments. A case series using dextrose/sucrose injections in 11 athletes with grade 3 anterior talofibular ligament injuries using between one and three injections showed a return to play mean of 62 days (range 38 to 87) and a similar mean for grade 2a anterior tibiofibular ligament injuries [56]. The athletes did not re-injure these ligaments subsequently, but the players who did not have prolotherapy but physiotherapy returned to play sooner.

Prolotherapy has also been advocated in syndesmotic ankle injuries.

## Plantar fascia

### Dry needling

**Fig. 9:**
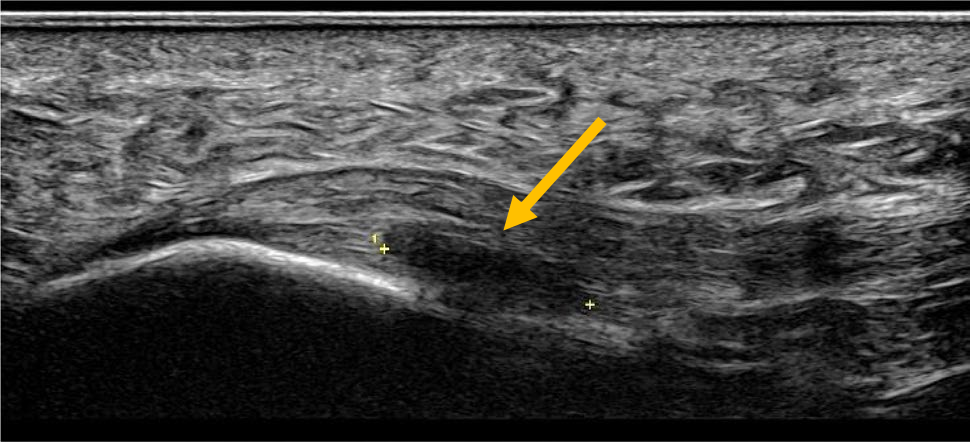
Ultrasound (longitudinal) showing a focal area of plantar fasciitis in the centre of the image (arrow)

**Fig. 10:**
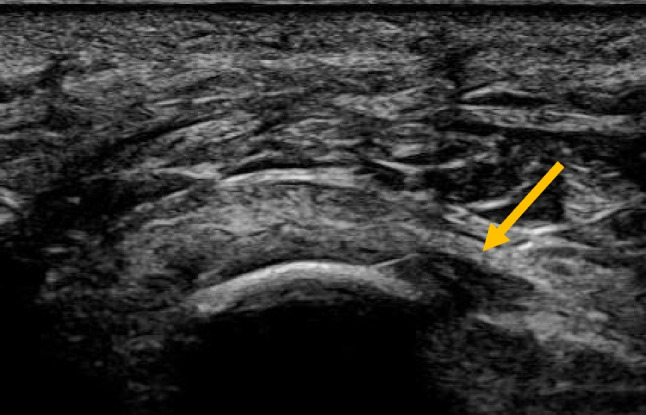
Ultrasound (Transverse) showing a focal area of plantar fasciitis on the right side of the image (arrow)

**Fig. 11:**
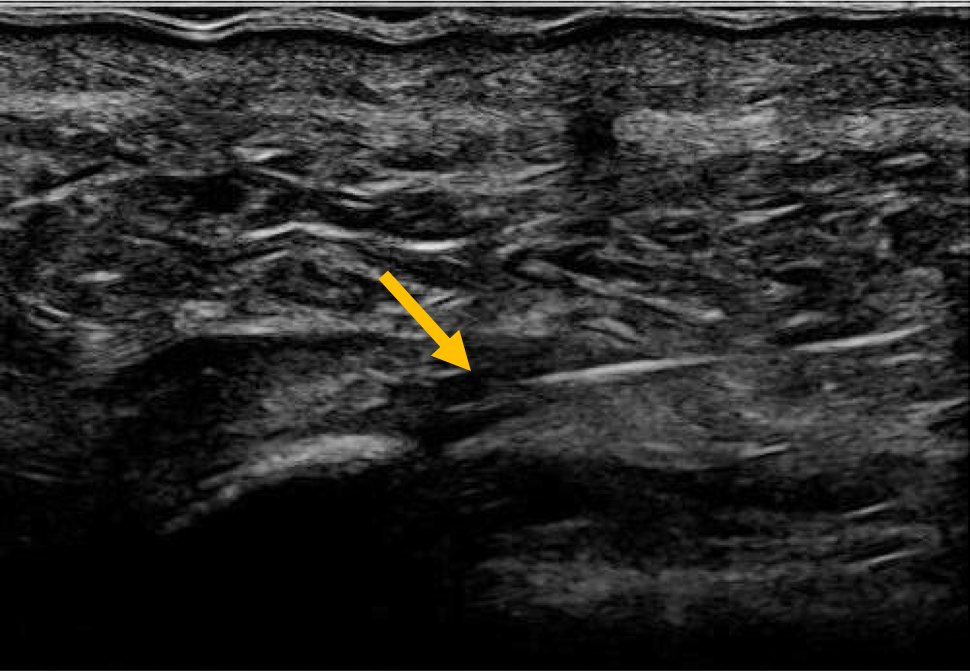
Ultrasound image showing dry needling of a focal area of plantar fasciitis in the centre of the image. The needle is at the side of the abnormality (arrow). The needle is going to penetrate the abnormality centrally

**Fig. 12:**
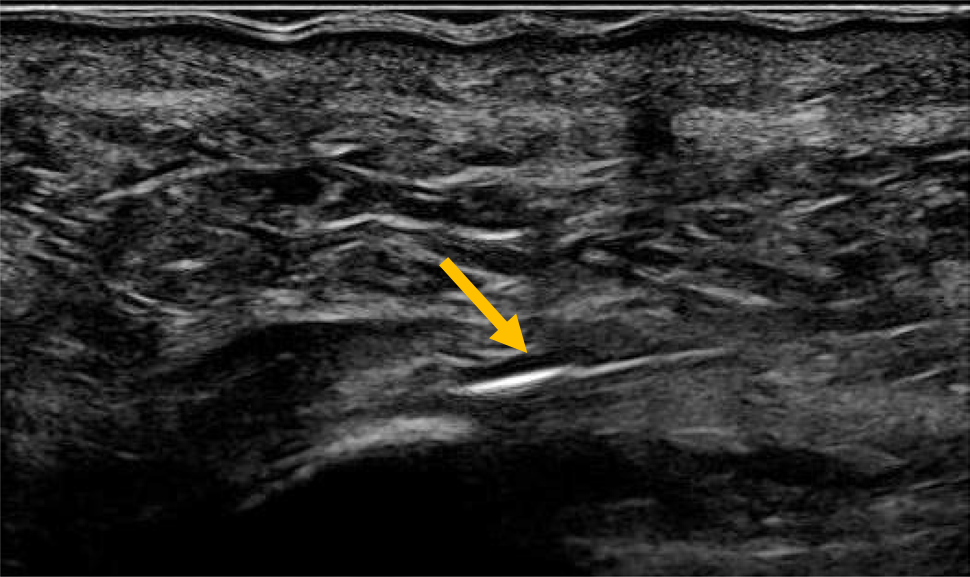
Ultrasound image showing dry needling of a focal area of plantar fasciitis in the centre of the image (arrow). The needle is inside the abnormality

Dry needling is used with good effect in this condition Figs. 9, 10, 11 and 12.

### PRP

PRP injections provide significant pain relief in chronic plantar fasciitis and have a better clinical outcome in the mid and long term compared to corticosteroid injections [51].

### Steroid

Gives good short-term pain relief, but this is at the risk of causing fat pad atrophy which can cause long-term pain.

MRI is not needed to assess for plantar fascia tears. Intervention in the plantar fascia should not be used in the acute phase of the tear. PRP with peppering is a useful treatment in established partial-thickness tears.

## After the procedure

Post-procedure advice should be given with the involvement of the physiotherapist, coach, and sports physician where relevant.

No procedure should be performed as a stand-alone treatment, and it is essential that all personnel involved in the player’s treatment be included in this advice.

If using steroids, a TUE (therapeutic use exemption) certificate needs to be completed, as the Football Association (FA) is bound by the World Anti-Doping Agency (WADA) rules. If the player is spot-checked, he could be banned from playing for 4 weeks.

## Conclusion

I have discussed the most relevant use of ultrasound intervention in elite soccer players, including the evidence, but also the experience of many years of providing injection therapies. This article was not meant to be exhaustive, and therefore, only the most common interventions have been discussed in detail. I hope this will stimulate the reader to be thoughtful when performing injection therapies and help with more evidence-based research in the future. Injection therapies are at the discretion of the interventionist as the evidence is poor in many areas and depends on the specific injury and the overall treatment goals.

All injection therapies should be used in addition to rehabilitation programmes. Injection therapy is primarily used in the acute phase after an injury to decrease pain and allow more function. There is some evidence that this will decrease the time a player “returns to play” as already discussed but return to training (RTT) and return to play (RTP) has to be assessed clinically to prevent further injury with imaging playing a small part in this decision [57–60].
